# Complications using tissue expanders in burn sequelae treatment at a reference university hospital: a retrospective study

**DOI:** 10.1590/0100-6991e-20202662

**Published:** 2021-06-04

**Authors:** LUIZ PHILIPE MOLINA VANA, RODOLFO COSTA LOBATO, JOÃO PAULO FONTANA BRAGAGNOLLO, CRISTIANE PEREIRA LOPES, HUGO ALBERTO NAKAMOTO, CARLOS FONTANA, ROLF GEMPERLI

**Affiliations:** 1 - Hospital das Clínicas - Faculdade de Medicina da Universidade de São Paulo, Departamento de Cirurgia Plástica e Queimaduras - São Paulo - SP - Brasil

**Keywords:** Tissue Expansion Devices, Tissue Expansion, Burns/Complications, Dispositivos para Expansão de Tecidos, Expansão de Tecido, Queimaduras/Complicações

## Abstract

**Background:**

tissue expanders have high relevance in plastic surgery and among indications it is worth mentioning their use in the treatment of burn reconstruction. Although it shows good results, its use requires special care because some complications can interrupt the reconstruction process. The objective of this study was to report the experience of the Clinics Hospital (University of Sao Paulo) with the use of tissue expanders to treat burn sequelae, establishing the incidence of complications, and identifying risk factors for their occurrence.

**Methods:**

a retrospective, observational, and analytical study, evaluating the use of expanders in burns sequelae treatment from 2009 to 2018.

**Results:**

245 expanders were placed in 84 patients, 215 were female, with a mean age of 19.96 years, being 40% in the trunk and 20% in the scalp, with a predominance of rectangular shape in 76.7% of cases. Complications were classified as major and minor.Complications occurred in 17.95% of cases, and extrusion and infection were the most common. There was a higher incidence of complications in expanders used in the upper and lower limbs as well as in those who did not undergo concomitant expansion (p <0.05), with an even higher chance of major complications in patients submitted to additional expansion. From 2009 to 2018, we observed a decrease in the incidence of complications.

**Conclusion:**

the complication rate (17.95%) is similar to other studies of the literature, there was a higher rate of complication with expanders placed in the limbs and a higher rate of major complications when additional expansion was done.

## RESUMO

**Introdução:**

os expansores teciduais são de importante relevância na cirurgia plástica, e dentre suas indicações destaca-se seu uso no tratamento de sequelas de queimaduras. Ainda que apresente bons resultados, seu uso requer cuidados especiais pois a incidência de complicações não é desprezível.

**Objetivo:**

relatar a experiência do Hospital das Clínicas da FMUSP com a utilização de expansores teciduais para tratamento de sequelas de queimaduras, estabelecendo a incidência de complicações e identificando possíveis fatores de risco para ocorrência das mesmas.

**Método:**

estudo retrospectivo, observacional e analítico, avaliando o uso de expansores no tratamento de sequelas de queimaduras no período de 2009 a 2018 no Hospital das Clínicas da FMUSP.

**Resultados:**

no período de análise estabelecido, foram colocados 245 expansores, sendo 215 em pacientes do sexo feminino, com idade média de 19,96 anos, sendo 40% em tronco e 20% em couro cabeludo, predominando uso do retangular em 76,7% dos casos. As complicações foram divididas em maiores e menores. As complicações ocorreram em 17,95% dos casos, sendo extrusão e infecção as de maior incidência. Houve maior incidência de complicação em expansores utilizados nos membros superiores e inferiores bem como naqueles que não realizaram expansão concomitante (p<0,05), havendo ainda maior chance de complicações absolutas nos pacientes submetidos à expansão adicional. Observou-se também diminuição no número de complicações entre 2009 e 2018.

**Conclusão:**

a taxa de complicação (17,95%) é semelhante a da literatura, observando-se maior taxa de complicação nos expansores colocados em membros e maior taxa de complicações absolutas quando realizada expansão adicional.

## INTRODUCTION

In the last 30 years, tissue expansion has become a well-established reconstruction modality to treat soft tissue defects^1^ ^-^ ^3^. Tissue expanders can be used to treat different conditions^4^ ^-^ ^6^, but scars resulting from burns stand out as one of the main indications of the expanders, once the presence of cicatricial retractions can lead to functional limitations of the cervical-mandibular region and limbs, as well as growth disorders^6^ ^-^ ^8^.

Despite the versatility of tissue expansion, it has a high incidence of complications, ranging from 20 to 40%, in the literature^6^ ^-^ ^10^. Some authors claim that these complications are more related to its use in children, in the lower limbs, in previously expanded areas and in patients with burn sequelae, and infection and extrusion are the most common complications^4^ ^,^ ^6^ ^-^ ^10^. These facts lead some surgeons to try avoiding the use of expanders, especially those who do not have such experience with them^8^ ^-^ ^10^.

The literature, so far, has not reported whether the complication rate varies when performed by residents of plastic surgery still in training.

## OBJECTIVE

To describe the epidemiological data and the incidence of complications with tissue expanders used to treat burn sequelae, from 2009 to 2018, in a university hospital (Hospital das Clinicas - Plastic Surgery Department - University of Sao Paulo), where all the operations were done by plastic surgery residents.

## METHODS

Patients’ medical records from the Burn Sequelae department were reviewed from January 2009 to December 2018, and patients who had undergone burn sequelae treatment with tissue expanders were included. This study was approved by Ethics Committee (CAPPesq) under the number 0084/10, and all patients were informed about the study and agreed their participation by filling the Consent Form. 

Data recorded were age, sex, expanded body segment, expander shape, expander volume, expansion period, previous expansion (additional expansion), use of more than one expander (concomitant expansion), the occurrence of complication, and types of complications.

Complications were classified as major and minor. We defined major complications as the ones that required early removing of the expander and those that led to complete failure of preoperative planning, and minor complications as the ones , those that did not require a new surgical intervention to save the expander and/or if the intervention was done, it did not lead to tissue expander removal, allowing the preoperative goal to be, at least, partially achieved^4^.

Surgical Procedure: since the Hospital das Clinicas is a university hospital, all operations were carried out by plastic surgery residents under direct supervision of an attending. Patients underwent the following standardized approach:

First surgery - Tissue expander placement: under general anesthesia, an incision was made, 1 mm away from the scar tissue in the healthy skin; the supra-fascial pocket created for the allocation of the expander was 1 cm larger in width and length than the expander dimensions; all cases had a closed negative pressure drain placed, which was removed after 5 to 7 days; the pocket closure was made in 3 or 4 levels with absorbable sutures, and an immediately expansion of 10% of the expander volume was done at the end of the procedure. Cephalexin and painkillers were prescribed for seven days.

After two weeks, the expansions begun, once a week, until the target volumes were achieved. To perform these expansions, the following routine was adopted: after rigorous antisepsis, a 0.9% saline solution was infused with a 10-20cc syringe and 25-gauge scalp needle; the infused volume for each expansion was based o’ the patie’t’s and flap’s tolerance, so it was stopped immediately when the patient reported discomfort and/or when the flap turned pale. All tissue expanders were expanded up to 1.5 to 2 times the original volume of the expander. Once achieving such volume, the removal of the expander and flap advancement occurred after two weeks. 

Second Surgery - Expander removal: For this procedure, the routine was: incision of the edge of the expanded area next to the scar to perform radial capsulotomy, place a negative pressure closed drain and close the flap in 3 levels with absorbable sutures.

All data underwent statistical analysis using the IBM® SPSS Statistics, version 23.0 (using the Likelihood Ratio Test for categorical variables and the Mann-Whitney and Kruskal-Wallis Tests for non-parametric variables). Values of p<0.05 were considered statistically significant.

## RESULTS

**Figure 1:**
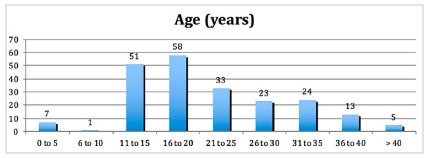
Number of tissue expanders by age.

From January 2009 to December 2018, 245 tissue expanders were placed in 84 patients who had undergone burn sequelae treatment. 215 (87.75%) were placed in females and 30 (12.24%) in males. The age of the patients ranged from 4 to 57 years (mean of 19.96 +/- 9.6 years), and the most common patients were 11 to 20 years old (109 - 44.5%) (Figure 1).

The trunk was the main body area where the expanders were placed (40%) and the rectangular one was the most used shape (76.73%). 45.31% were primary expansions and the other 54.69% of the cases had already undergone previous expansions. 97 expanders were used concomitantly, whereas 148 were used alone (Table 1).

**Table 1:** The anatomical expansion sites, tissue expander shape, previous, and concomitant expansion.

Site	Number	Percentage
Scalp	49	20%
Head and neck	48	19.6%
Trunk	98	40%
Upper limbs	40	16.3%
Lower limbs	10	4.1%
Tissue expander shape	Number	Percentage
Rectangular	188	76.73%
Croissant	30	12.24%
Round	12	4.89%
Longitudinally curved	15	6.12%
Previous expansion	Number	Percentage
Yes	134	54.69%
No	111	45.31%
Concomitant expansion	Number	Percentage
Yes	97	39.59%
No	148	60.41%

Complications happened in 44 expanders (17.95%): 25 major complications (10.20%) and 19 minor complications (7.75%). The most prevalent major complications were extrusion of the implant (10 cases - 40%) and infection (9 cases - 36%), while the most common minor complication was dehiscence (12 cases - 63,15%). (Table 2).

**Table 2:** Tissue expander complications.

Complications	Number	Percentage
Yes	44	17.95%
No	201	82.05%
Type of complication	Number	Percentage
Major	25	10.20%
Minor	19	7.75%
Type of major complication	Number	Percentage
Extrusion	10	4.09%
Infection	9	3.69%
Roture	3	1.23%
Valve problem	3	1.23%
Type of minor complication	Number	Percentage
Dehiscence	12	4.88%
Infection	5	2.05%
Hematome	1	0.41%
Valve problem	1	0.41%

Regarding the reconstruction procedures, among 220 patients (201 without complications and 19 with minor complications), flap advancements were carried out in 216 cases (88.16%) and 4 tissue expanders (1.64%) were used to expand the latissimus dorsi muscle flap in order to do breast reconstruction.

Table 3 has registered the expansion sites that can have some influence on complication rates, with a higher incidence of complications in expanders that were positioned in upper and lower limbs (p <0.05). Furthermore, cases without concomitant expansion had a higher complication rate (p <0.05). 

**Table 3:** Epidemiological data and the occurrence of complications (patients with no Major complications - 220 tissue expanders).

Variable	Category		Complication (y/n)				Sig.(p)
		Total	Yes		No		
		Number	Number	Percentage	Number	Percentage	
Gender	Male	30	4	13.30%	26	86.70%	0,481
	Female	215	40	18.60%	175	81.40%	
Age	< 20	141	29	20.60%	112	79.40%	0,145
	20-40	99	13	13.10%	86	86.90%	
	> 40	5	2	40.00%	3	60.00%	
Site	Scalp	49	13	26.50%	36	73.50%	0,000
	Head and neck	48	6	12.50%	42	87.50%	
	Trunk	57	4	7.00%	53	93.00%	
	Abdomen	41	3	7.30%	38	92.70%	
	Upper limb	40	14	35.00%	26	65.00%	
	Lower limb	10	4	40.00%	6	60.00%	
Tissue expander shape	Retangular	188	32	17.00%	156	83.00%	0,463
	Semi-lunar	30	5	16.70%	25	83.30%	
	Round	12	2	16.70%	10	83.30%	
	Long. curved	15	5	33.30%	10	66.70%	
Additional	Yes	134	24	17.90%	110	82.10%	0,983
	No	111	20	18.00%	91	82.00%	
Concomitant	Yes	117	13	11.10%	104	88.90%	0,008
	No	128	31	24.20%	97	75.80%	

Table 4 indicates the association between major and minor complications with the studied variables. Additional expansion is associated with a higher frequency of major complications (p <0.05).

**Table 4:** Epidemiological data and the occurrence of major and minor complications.

Variable	Category		Type of complication				Sig. (p)
		Total	Major		Minor		
		Number	Number	Percentage	Number	Percentage	
Gender	Male	4	3	75.00%	1	25.00%	0,441
	Female	40	22	55.00%	18	45.00%	
Age	< 20	29	17	58.60%	12	41.40%	0,940
	20-40	13	7	53.80%	6	46.20%	
	> 40	2	1	50.00%	1	50.00%	
Site	Scalp	13	10	76.90%	3	23.10%	0,173
	Head and neck	6	3	50.00%	3	50.00%	
	Trunk	4	2	50.00%	2	50.00%	
	Abdomen	3	2	66.70%	1	33.30%	
	Upper limb	14	8	57.10%	6	42.90%	
	Lower limb	4	0	0.00%	4	100.00%	
Tissue expander shape	Retangular	32	16	50.00%	16	50.00%	0,356
	Semi-lunar	5	3	60.00%	2	40.00%	
	Round	2	2	100.00%	0	0.00%	
	Long. curved	5	4	80.00%	1	20.00%	
Additional	Yes	24	17	70.80%	7	29.20%	0,040
	No	20	8	40.00%	12	60.00%	
Concomitant	Yes	13	7	53.80%	6	46.20%	0,797
	No	31	18	58.10%	13	41.90%	

## DISCUSSION

Tissue expanders have been used by the Department of Plastic Surgery and Burns at the Hospital das Clínicas, University of São Paulo, since 1984^11^ ^-^ ^13^. Looking for an updated epidemiological characteristics of such patients, treated by the plastic surgery residents, in the 2009-2018 period, it was observed a higher frequency of tissue expanders in women (87.75%) and in patients between 11 and 20 years old (44.5%). Cunha et al.^14^, Fochtmann et al.^15^, and Yeong et al. 16 had also found the same trend. Almeida et al.^17^ and Nakamoto et al.^18^ attributed these findings to the fact that this group of patients seeks better aesthetic results, which is frequently achieved by the use of expanders. 

Most of the expanders in this case series were placed in the trunk (40%), and the most common shape was rectangular (76.73%), similarly to Bozkurt et al.^5^ and Yeong et al.^16^. Both authors, therefore, diverge from Fochtmann et al.^15^ and Nakamoto et al.^18^, who found that most of the expanders were placed in lower limbs and scalp, respectively. Cunha et al.^14^ placed an equal number of expanders in both trunk and scalp. There is no agreement regarding which is the most popular site for tissue expansion placement. However, we should highlight that scalp and trunk are generally pointed out as the most common sites for tissue expander placement in burns, probably because these areas respond better to expansion, especially the scalp, which has a low elasticity for large local flaps and there is no need to make hair transplantation if the scalp flap is used.^15^ We must also mention that the profile of burns is important to explain the demands of patients.

Considering the shape of the expander, Cunha et al.^14^ observed a similar amount of semi-lunar and rectangular expanders, diverging from our data, in which rectangular expanders were predominantly used (76.73%). Yeong et al.^16^ also used rectangular expanders in 88% of cases, and so did Bozkurt et al.^5^, who placed rectangular expanders in 48% of their patients. The reason for that is the fact that rectangular expanders are indicated in large areas, therefore, it is used in burned patients who demand more extensive resections of scars. However, the availability of the material can vary from center to center, which may end up influencing this choice.

In this study, the total complication rate was 17.95%, with major complications - 10.2% and minor as 7.75%. When we compare our data to Bozkurt’s et al.^5^, their incidence of complications was 28.4%, dividing them into -minor - 18.6% (hematoma, seroma, and delayed healing) and -major - 9.8% (infection, extrusion, leakage, dehiscence, and cutaneous necrosis). Elshahat et al.^19^ had 16.6% complication rate and 6.6% were classified as absolute, leading to complete failure of the proposed treatment. Yeong et al.^16^ presented a much higher complication rate, with an incidence of 53% (33 out of 62 cases), 15% of which were absolute complications. We can s’e that Yeong’s study presented an incidence of complications higher than ours, with twice as many absolute complications. The authors justify this high incidence with the following arguments: 1) they used expanders only in burn sequelae treatment (not for other disease states), 2) their patients had an extensive burned body surface (mean of 40% SCQ) - which would influence the worse quality of cutaneous tissue and could lead to a greater chance of complications, such as dehiscence^20^, and 3) the great majority of the expanders were placed in the head and neck region, near the mandible, fact that could facilitate extrusion due to the thin skin and jaw movement^16^.

Cunha et al.^14^ also in a retrospective study at Hospital das Clinicas - São Paulo, evaluating the complication rate with the use of tissue expanders over 10 years (from 1991 to 2000), not only for burn sequelae treatment, showed a total complication rate of 22.2%, of which 19.3% were absolute complications and 2.9% were relative. Fochtmann et al.^15^ also evaluated the incidence of complications with the use of expanders without distinction of patient groups, observing 33% of total complications (in 49 of the 148 expands placed), 21% of which were absolute, and 12% were relative complications. 

As seen in the literature, complication rates while using tissue expanders for the treatment of burn sequelae differ widely, ranging from 7.5%^21^ to 45%^22^, while in pediatric burn patients, these rates range from 9% to 37%^23^. However, most of the recent studies show lower rates, ranging from 15% to 25%, considering only those applied to the treatment of burn sequelae. This distinction is vital since burn sequelae patients usually have an extensive area of scar and a higher chance of dehiscence, which may justify a relatively higher incidence of complications^20^. We have summarized in Table 5 the complication rates seen in various studies.

**Table 5:** Comparison of the main studies and their complications rates.

	Assessed period (years)	Number of patients	Number of expanders	Indication	Site of expansion	Major complication	Minor complication	Statistical analysis
Pisarski^23^	11	301	403	Burn sequelae	Multiple sites	11%	7.1%	N/A
Cunha^14^	10	164	315	Multiple etiologies	Multiple sites	19.3%	2.85%	T Student
Patel^29^	10	240	256	Burn sequelae	Multiple sites	14.1%	10.2%	Pearson independent /Chi-squared Test
Current study	10	84	245	Burn sequelae	Multiple sites	10.2%	7.75%	Mann-Whitney, Kruskal-Wallis, LR Test
Fochtman^15^	17	73	148	Burn sequelae X Multiple etiologies	Multiple sites	21%	12%	Odds Ratio
Lopez^25^	30	73	141	Burn Sequelae	Lower limbs	18.4%	10.6	N/A
Pandya^24^	8	88	113	Multiple etiologies	Limb X No limb	L= 17% X NL=14%	L= 26% X NL= 13%	N/A
Bozkurt^5^	9	57	102	Burn sequelae	Multiple sites	9.8%	18.6%	Chi-squared test
Yeong^16^	8	37	62	Burn sequelae	Multiple sites	14%	39%	Multiple logistic regression analysis
Elshahat^19^	4	53	60	Burn sequelae	Multiple sites	6.6%	10%	Chi-squared test
Tavares Filho^28^	19	23	54	Burn sequelae	Multiple sites	7.5%	15%	N/A
Saleh^26^	3	40	40	Multiple etiologies	Scalp	13.25%	21.5%	N/A
Ashab Yamin^30^	1	36	43	Burn sequelae	Head and neck	13.89%	2.78%	N/A
Tavares Filho^27^	14	17	24	Multiple etiologies	Lower limb	20.9%	16.7%	Fisher
Bjornson^31^	10	24	93	Multiple etiologies	Multiple sites	20.4%	Not analyzed	Fisher

Complications reported in the literature mainly include infection, expander exposure, and failure at the time of expansion. Other complications are injection port malfunction, local pain, hematoma, seroma, bone resorption and enlarged scars^10^ ^-^ ^14^. In the present study, the incidence of extrusion and infection was almost the same (10 and 9 cases, respectively), but the most common among all was dehiscence (12 cases), considered a minor complication, once all the cases could be saved (Table 2). The most recent studies also had infection and extrusion as the most frequent complications^5^ ^,^ ^15^ ^,^ ^19^ ^,^ ^31^.

Table 3 shows that expansion site influences complications rates, with a higher incidence of complications in expanders positioned in upper and lower limbs (p <0.05), as found by Bozkurt et al.^5^ and Elshahat et al.^19^ (lower limbs is the site where complications happen more frequently and have higher rates of reconstruction’s failures). Pandya et al.^24^ have provided some explanations regarding why the expansion in upper and lower limbs would have a higher incidence of complications: difficulty in creating the pocket for the expander in the extremities; frequent mobilization of the limb with the expander, which could increase the pressure on the created pocket; the presence of an incision near the expanded area, facilitating its dehiscence. 

Table 3 also shows that, in cases where concomitant expansion was not performed, the incidence of complications was higher (p<0.008), which is counterintuitive, because we tend to imagine that there are more complications when using more than one expander simultaneously. We believe that the incidence of complications was higher in the group that we used only one tissue expander because they were patients with lesions in more critical areas, presenting thin skin with intense fibrosis, and were more challenging to expand. So, these conditions of the tissue would not allow the use of two or more expanders simultaneously, leading to higher complication rates, even when using only one tissue expander. Further observations and evaluation of the type of patient, exact location, as well as local characteristics, are necessary to clarify this finding. Since most studies do not address concomitant (or simultaneous) expansion, no data were found to corroborate or disagree with our data. 

Table 4 presents that additional expansion was associated with a higher frequency of major complications (p <0.04), but Fochtmann et al.^15^ reported opposite results, as the author point out, the risk of complications using expanders is reduced by 0.995 times for each additional (sequential) expander placed in the same patient but without statistical significance. Cunha et al.^14^ and Bozkurt et al.^5^ did not observe any difference between these groups.

In this study, we observed that the fact that our surgeries were performed by residents of plastic surgery in training supervised by an attending physician, did not increase the complication rates in the use of tissue expanders in the treatment of burn sequelae, keeping them even below that of many studies analyzed (Table 5).This study has the limitation of being a retrospective analysis and we do not have statistical data to support some routines used, but we believe that their systematization helps to reduce the incidence of complications. In this way, we suggest the degermation of the tissue expander site, a small incision distancing 1mm from the healing skin, a store slightly larger than the size of the expander, always use a negative pressure drain, closure of the bag with 3 to 4 layers and antibiotics for seven days.

## CONCLUSION

This study allows us to conclude that patients with burn sequelae treated with tissue expanders at the Hospital das Clínicas, University of Sao Paulo between 2009 and 2018 had similar epidemiological characteristics to those reported in the literature (usually women, young people with expanders placed mainly in the trunk and rectangular shape) and an acceptable general complication rate (17.95%), below the literature (27.78%). We also observed that the placement of the expanders in the upper and lower limbs could be considered a risk factor for the occurrence of complications and that concomitant tissue expansion may not increase the chances of absolute complications.
